# Sex and gonadal hormones in mouse models of Alzheimer’s disease: what is relevant to the human condition?

**DOI:** 10.1186/2042-6410-3-24

**Published:** 2012-11-05

**Authors:** Dena B Dubal, Lauren Broestl, Kurtresha Worden

**Affiliations:** 1 Laboratory of Neuroscience and Aging Research, Department of Neurology, Sandler Neurosciences Center, Room 212B, University of California, San Francisco, San Francisco, CA 94158, USA

**Keywords:** Sex, Gender, Brain, Alzheimer’s disease, Aβ, Tau, Neurodegeneration, Sex chromosomes, Hormones, Estrogen, Progesterone, Testosterone, Androgens, Cognition, Behavior, Pathology, Transgenic, Mouse, Human, Aging, Reproductive aging, Menopause, Andropause

## Abstract

Biologic sex and gonadal hormones matter in human aging and diseases of aging such as Alzheimer’s – and the importance of studying their influences relates directly to human health. The goal of this article is to review the literature to date on sex and hormones in mouse models of Alzheimer’s disease (AD) with an exclusive focus on interpreting the relevance of findings to the human condition. To this end, we highlight advances in AD and in sex and hormone biology, discuss what these advances mean for merging the two fields, review the current mouse model literature, raise major unresolved questions, and offer a research framework that incorporates human reproductive aging for future studies aimed at translational discoveries in this important area. Unraveling human relevant pathways in sex and hormone-based biology may ultimately pave the way to novel and urgently needed treatments for AD and other neurodegenerative diseases.

## Introduction

Biologic sex and gonadal hormones exert profound effects on brain function – and we are only beginning to appreciate the complexities of their actions in Alzheimer’s disease (AD) from studies of humans and mouse models. In 2010, the Institute of Medicine advocated for expansion of neuroscience research to understand sex differences in the susceptibility and progression of key neurodegenerative conditions such as AD [1]. Indeed, the importance of delineating sex- and hormone-based actions in AD cannot be underestimated for many reasons. First, AD is a tragic disease and the most common neurodegenerative condition, characterized by an insidious and progressive loss of memory and other cognitive functions. Second, true sex-based differences in AD exist. Thus, a more clear understanding of the exact nature of the sexual dimorphisms can shed light on what protects one sex or makes the other more vulnerable. Third, AD develops in an aging brain and a fundamental aspect of human aging is gonadal steroid depletion. Whether and how depletion of certain androgens in men and estrogens and progestins in women can affect brain health and vulnerability to AD emerge as highly relevant, and still unanswered, clinical questions. Finally, sex- and hormone-based actions in human AD lay the groundwork for the intelligent design, execution, and interpretation of studies in animal models of aging and AD. Ultimately, animal models of aging and disease enable rigorous dissection and mechanistic delineation that may pave the way to novel and urgently needed treatments to defeat AD.

In this Review, we highlight advances in AD, describe and interpret sex- and hormone-based studies of AD, and discuss the importance of simulating human reproductive aging when modeling diseases of aging. With the human condition in mind, we then review mouse models of AD, analyze reports of sex differences and hormone effects in male and female mice that model AD, raise major unresolved questions, and offer a research framework that incorporates human reproductive aging for future studies aimed at translational discoveries in the important area of sex and hormone biology.

## Alzheimer’s disease

### AD

Alzheimer’s disease, the most common neurodegenerative condition, is reaching epidemic proportions. In the absence of effective interventions, over 50 million people worldwide will suffer from this devastating dementia by the year 2050 [2]. The symptoms of AD begin insidiously with memory impairment and then gradually progress to erode multiple cognitive and behavioral functions. The immeasurable burdens of the disease, combined with a history of failed clinical trials (reviewed in [3,4]) warrant urgent action toward the development of novel therapeutic targets based on a deeper understanding of AD.

### Cognitive decline in AD

Progress in multiple fields of human and mouse model research has advanced our knowledge of what leads to cognitive decline in AD (for full review [5]). We now know that synaptic loss [6-8] and network dysfunction [9,10] correlate more closely with cognitive deficits in AD than neuronal loss and degeneration. Furthermore, we have a growing appreciation based on imaging findings [11-13] and pathology studies [14-17] that the burden, distribution, or presence of amyloid plaques, pathologic hallmarks of AD, do not correlate well with cognitive dysfunction. These human observations, combined with evidence from transgenic mouse models of AD, also support the concept that plaques and neurofibrillary tangles, though potentially toxic in their own right [18-20], may not be the primary or driving cause of cognitive dysfunction. Highlights from this large body of literature include: cognitive deficits often develop prior to the deposition of amyloid plaques [21-23], neurofibrillary tangles can exist without neuronal impairment [14,16,24-27], and tau alone can exert toxicity independently of neurofibrillary tangles (reviewed in [28]). Thus, a growing body of literature suggests that synaptic loss and dysfunction and network disruptions, rather than conventional pathological hallmarks, are main players in the development of cognitive decline in AD.

### Multifactorial etiologies of AD

AD is a complex disease caused by the interaction of many factors. Aging, itself, is the primary risk factor for the development of AD and aging–related problems such as diabetes, hypertension, and hyperlipidemia may further promote AD risk [29]. Genetic contributions to AD include mutations or alleles that increase risk such as ApoE4 [30-33] and GWAS-identified genes [30,34] or decrease risk such as the A673T coding variant in *APP*[35]. It is worth noting that, to date, all familial AD cases have been caused by either mutations, duplications, or overexpression, of the human amyloid precursor protein (hAPP) or by mutations in presenelin 1 (PS1) or presenelin 2 (PS2), which alter the processing of hAPP (reviewed in [36]).

The human genetics of AD, combined with several lines of evidence in human and mouse studies demonstrate a pathogenic role for Aβ, and particularly for soluble, oligomeric assemblies of Aβ in synaptic and network dysfunction [10]. Aβ can alter and depress synaptic function through mechanisms that involve NMDAR trafficking [37], tau mislocalization into dendritic spines [38,39], and a host of other mechanisms (reviewed in [10,40]) that may ultimately lead to network destabilization and cognitive dysfunction (reviewed in [9,10,40]).

## AD: sex^a^ and epidemiology

### Epidemiology^b^

Alzheimer’s disease and mild cognitive impairment (MCI), a clinical state preceeding AD, affect men and women in different ways. A thorough understanding of the sex-based differences in prevalence, incidence, and disease course can provide critical insight into potential targets for prevention. Of note, our review focuses on large-scale epidemiologic studies, which do not often specify effects of sex-influenced risk factors such as ApoE4, an important modifier of AD [5].

**Figure 1:**
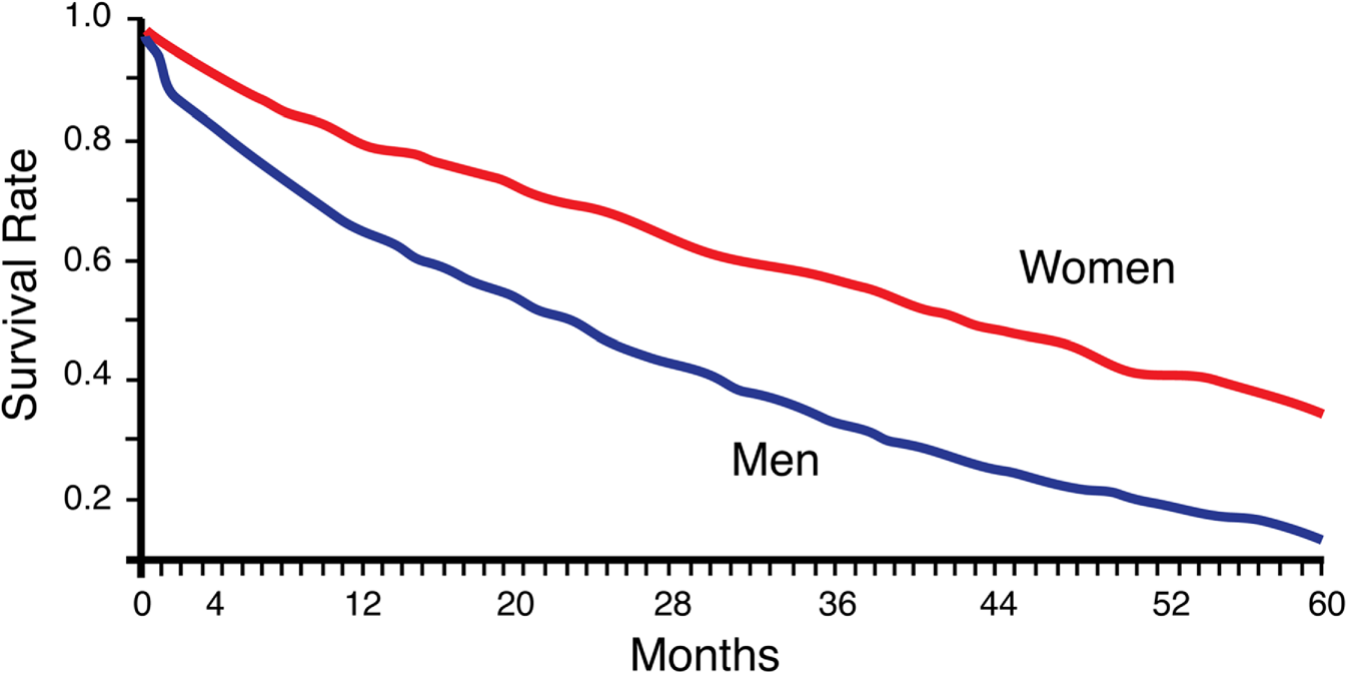
**Males with AD progress to death faster than females.** Survival rate in an AD cohort of age-matched men and women shows that males progress faster to death with sporadic AD in a manner that correlates most closely with cognitive decline. This effect is observed in sporadic [44,45] and early-onset AD [41-43]. Figure adapted from [45] with permission from *Neurology*.

Here, we review epidemiologic data on AD with an emphasis on an important yet underappreciated sexual dimorphism: women bear a greater burden of AD due to increased prevalence and possibly incidence, but men suffer an aggressive course of the disease (Figure 1) [41-45]. In fact, one of the strongest predictors for an aggressive disease course and progression to death following a diagnosis of AD is male sex [44].

### Prevalence

The prevalence of AD, or the total number of cases in a population at a given time, is higher in women compared to men in multiple populations [46]. This is due, in large part, to female longevity – that is, women are more likely to live to ages when AD is most prevalent. In contrast to AD, the prevalence of mild cognitive impairment (MCI), a cognitive state that precedes dementia, is higher in men in many populations [47-49], although not all studies are in agreement [50,51]. Together, these data suggest that men may be more vulnerable to the onset of the disease.

### Incidence

The incidence of AD, a measure of the risk of developing disease over time, is on the whole similar between men and women. Many epidemiologic studies show increased risk for the development of AD in women compared to men in specific populations, and many do not. A meta-analysis of studies worldwide shows similar risks between men and women that increase dramatically with age, and may increase disproportionately in women after the age of 80 yrs [52]. In parallel with increased prevalence of MCI in men, the Mayo Clinic Study of Aging also shows increased risk of MCI (or incidence rates) in men [53]. It will be important to see if other studies show the same.

### Disease Course

Sexual dimorphism in the progression of AD is a major and meaningful epidemiologic measure that has received very little attention compared to incidence and prevalence. Men are more vulnerable to an aggressive disease course compared to women. This underappreciated sex difference is supported by several studies. First, men progress to death faster than women in both early- [41-43] and late-onset [44,45] AD (Figure 1). Since the sex difference exists in the presence and absence of other age–related comorbidities like cancer and heart failure, it suggests increased vulnerability to the pathophysiology of AD in men compared to women. In addition, the observed progression to death closely correlates with the rate of cognitive decline [54]. In further support of a more aggressive course of AD in men, more studies are finding increased MCI in men [47-49,53,55], suggesting increased vulnerability to the development and manifestation of cognitive deficits.

### AD: Disease course and mouse models

Disease course may be the human epidemiologic factor most relevant to our study of AD in animal models. Since most mouse models of AD involve the transgenic expression of mutated APP with or without mutated tau, outcome measures are focused squarely on the manifestation or disease course rather than the risk or prevalence of disease. Specifically, mouse models enable study of how a manipulation changes the manifestation or severity of AD–related disease measures such as pathology, biochemistry, cognition/behavior, synaptic/network plasticity, or survival.

## Human reproductive aging, hormone replacement and AD

### Reproductive Aging

**Figure 2:**
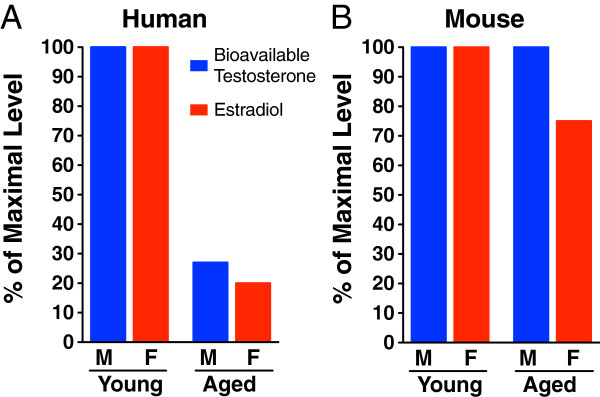
**Gonadal hormone decline occurs in human, but not mouse, reproductive aging.** (**A**) Human levels of bioavailable testosterone (blue) in males and estradiol (the most biologically active estrogen) (red) in females decrease dramatically during aging at a time when the brain is vulnerable to neurodegenerative diseases such as AD. Of note, changes in total testosterone (not shown) are more modest. (**B**) In contrast with the human condition, mouse levels of bioavailable testosterone (blue) and estradiol (red) do not decrease with age. M=male and F=female. Data derived from human [56-60] and mouse [61,62] studies.

Reproductive aging is a fundamental aspect of the aging process and is accompanied by dramatic decreases in certain gonadal steroid levels in human [56-60], but not rodent [61-64] males and females (Figure 2). Since AD is a disease of the aging brain and develops in a sex hormone-depleted environment, it is critical to study how gonadal steroid changes in menopause and andropause affect the risk and course of AD in women and men. Further, simulation of reproductive aging in mouse models of AD via gonadal steroid depletion represents a meaningful way to model the human condition.

### Women, AD, and Hormone Replacement Therapy (HRT)

The results of randomized, controlled clinical trials, including the Women’s Health Initiative (WHI) [65,66], found that HRT in women led to adverse [65-67] or no effects [68-70] on cognition or AD risk. These studies represent rigorous clinical trials; however, certain caveats such as hormone formulation, timing of therapy, and dose or route of hormone administration should be considered when interpreting the data (as reviewed in [71,72]). Ongoing [73] and future clinical studies should dissect whether HRT may be beneficial if given 1) during a “critical window”^d^ after menopause, 2) by subcutaneous routes which largely bypass the liver, 3) in lower doses, 4) or in other formulations since conjugated equine estrogens without medroxyprogesterone acetate (Premarin) or with it (Prempro) may have differing biological activities from hormones typically used in animal studies (estradiol and progesterone). While future studies may shed light on these complex possibilities, the clinical data to date show that HRT, in its current forms, can be deleterious to cognitive outcomes.

Because AD pathophysiology begins and progresses years before its clinical manifestation [74,75], it is conceivable that HRT accelerated an existing disease process in women who experienced cognitive decline in clinical trials. Along the same lines, it is also possible that HRT benefits mood and cognition in the context of normal aging, but not in women already at risk for developing AD. As we move closer to personalized medicine, the use of genetic, protein, and imaging biomarkers to predict healthy brain aging versus increased risk for AD and other diseases should serve as a clinical guide to whether HRT is appropriate for an individual woman.

### Men, AD, and Androgen Replacement

Andropause is the male correlate of the female menopause, characterized by a gradual but steady decline of certain circulating gonadal steroids. A modest decrease in total testosterone accompanied by an increase in steroid hormone binding globulin (SHBG) results in a major decrease of bioavailable testosterone, averaging 1-2% per year beginning in the third or fourth decade of life [58,60,76-79]. Bioavailable testosterone (testosterone not bound to SHBG) has long been recognized as the biologically active, and thus most critical form of androgen.

Low levels of biologically active testosterone in the aging male may be deleterious to the brain. Despite new findings that SHBG might facilitate steroid delivery to target tissues (reviewed in [80,81]), both increased SHBG [82-84] and low levels of androgens (reviewed in [85]) are associated with increased dementia and AD risk. These observations, combined with studies of androgen treatment in humans and mice, suggest a protective role for androgens in cognition and AD (reviewed in [85]). Rigorous clinical trials to determine whether androgen replacement is indeed beneficial to cognition and dementia risk are needed.

### Simulating human reproductive aging in mouse models of AD

An understanding of human AD epidemiology and reproductive aging, combined with intelligent research strategies to study effects of sex and hormones, set the stage to simulate the human condition using mouse models of AD. Both mice and humans undergo reproductive aging and subsequent decline in fertility. However two main differences in reproductive aging exists between the species. First, gonadal steroid levels decline in males and females during human aging [56-60] but not mouse aging [61-64] (Figure 2). Second, the primary cause of reproductive senescence in women is declining oocyte number and ovarian function [86]; in female rodents it is dysregulation of the neuroendocrine system [87,88]. Despite these differences, the unifying similarity in reproductive aging processes is that changes at all levels of the hypothalamic-pituitary-gonadal axis are important in both humans and rodents [62,87,89].

Simulation of a prominent aspect of human reproductive aging in mice can be accomplished by gonadal steroid depletion through gonadectomy or other methods [90]. Since AD develops in the aging human brain, which is subject to effects of sex hormone depletion in both sexes, gonadectomy in mouse models of AD recapitulates a critical aspect of human reproductive aging in males and females.

## Strategies for studying hormone and sex effects in mouse models of AD

### Addressing human-relevant questions

Since aging is the primary risk factor for AD and development of AD is restricted to the aging brain, the study of sex and hormones in light of reproductive aging, steroid depletion, and hormone replacement represents a translational framework for human-relevant research in mice. With this framework in mind, we propose three research areas, offer optimal research approaches using mice, and consider caveats. Each human-relevant question posed is followed by an optimal research approach using mice.

**Figure 3:**
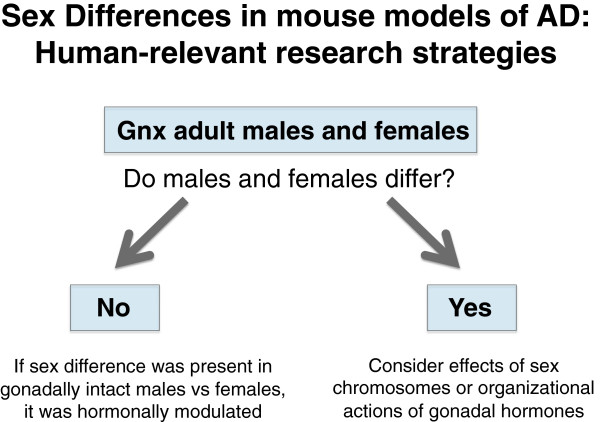
**Research strategy for studying sex differences in mouse models of AD.** Gonadectomy of both male and female mice simulates gonadal steroid depletion that occurs in human reproductive aging. It also enables a direct comparison between the sexes that is less confounded by differential, activational effects of gonadal hormones in the male versus female brain.

1. **Gonadal hormone depletion.** The following questions focus on sex differences (Figure 3).

a. Do males and females differ in AD-related outcome measures? Compare gonadectomized male and female mice to investigate sex differences.

b. If males and females differ in the absence of gonadal steroids, is the sex difference due to sex chromosomes or organizational effects of gonadal hormones? Delineate these possibilities using a genetic approach such as the “four core genotypes” (FCG) model (reviewed in [91,92]).

**Figure 4:**
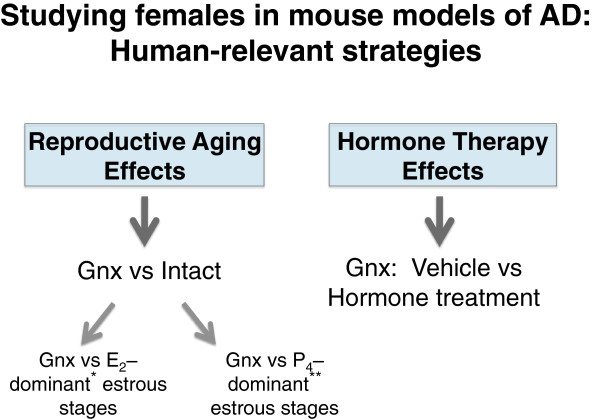
**Research strategy for studying females in mouse models of AD.** To determine how reproductive aging alters AD-related measures in females, an appropriate strategy is comparing gonadectomized to intact mice. Intact females can be separated into estrogen dominant* (E_2_: proestrous, estrous) or progesterone dominant** (P_4_: metestrous, diestrous) stages of the reproductive cycle since these hormones can have opposing effects in the brain. To determine whether hormone replacement alters AD-related measures in females, an appropriate strategy is comparing vehicle- versus hormone-treated mice that have all undergone gonadectomy to simulate human reproductive aging. E_2_ is estradiol, the most biologically active estrogen in the mammalian reproductive cycle.

**Figure 5:**
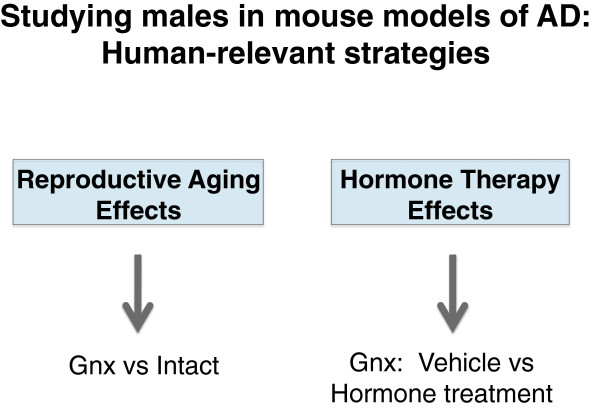
**Research strategy for studying males in mouse models of AD.** To determine how reproductive aging alters AD-related measures in males, an appropriate strategy is comparing gonadectomized to intact mice. To determine whether hormone replacement alters AD-related measures in males, an appropriate strategy is comparing vehicle- versus hormone-treated mice that have all undergone gonadectomy to simulate human reproductive aging.

2. **Reproductive aging.** The following questions focus on effects of reproductive aging.

a. How does reproductive aging alter vulnerability to AD in females (Figure 4)? Compare “intact” to gonadectomized females. Stage of estrous cycle may also be taken into account since estrogen and progesterone, which can have opposing actions, fluctuate.

b. How does reproductive aging alter vulnerability to AD in males (Figure 5)? Compare “intact” (gonads present) to hormone-depleted (gonadectomized) male mice.

3. **Hormone replacement.** The following questions focus on effects of HRT.

a. How does hormone replacement in gonadal steroid-depleted females alter vulnerability to AD? Compare vehicle vs hormone treatment in hormone-depleted (gonadectomized) female mice (Figure 4). HRT doses, formulations, regimens, timing, and routes of administration can be tested, as guided by clinical questions.

b. How does hormone replacement in gonadal steroid-depleted males alter vulnerability to AD? Compare vehicle vs hormone treatment in hormone-depleted (gonadectomized) male mice (Figure 5).

### Gonadectomy: considerations, caveats, and alternatives

All three areas of research outlined above incorporate the strategy of depleting gonadal hormones though gonadectomy to mimic an aspect of human reproductive aging. This manipulation successfully depletes levels of gonadal hormones, but a few points regarding its limitations and optimal applications deserve consideration. First, gonadectomy decreases hormone levels in a subacute, rather than a slow and progressive manner, as occurs in human reproductive aging. However, whether the rate of hormone decline influences AD-related outcomes is unknown. Second, gonadectomy performed during middle-age (10–15 months in mice) may more closely model reproductive aging, since central nervous system changes that underlie reproductive aging have already occurred; but restricting studies to aging mice is not always practical or economically feasible. Finally, gonadal hormone depletion through natural or surgical means in humans [56,93-96] or gonadectomy in mice [97,98] results in compensatory neuroendocrine responses such as increased luteinizing hormone (LH) and follicle stimulating hormone (FSH). Therefore, potential effects of LH and FSH cannot be ruled out when interpreting effects of gonadal hormone depletion. Nonetheless, since these compensatory changes occur in both simulated and true reproductive aging, the manipulation of gonadectomy in mouse models remains relevant to the human condition.

Alternatives to gonadectomy in female mice include genetic and chemical models of reproductive aging. The follitropin receptor knockout (FORKO) mouse is a genetic model that leads to chronic estrogen deficiency early in development [99,100]. An advantage of genetic models such as FORKO is the ability to recapitulate specific physiological mechanisms involved in human reproductive aging; however, limitations such as potential effects of the mutation on brain development and function could introduce confounds or constrain study interpretations. A chemical model of reproductive aging is treatment with 4-vinylcyclohexene diepoxide (VCD), an industrial chemical that induces follicle depletion and ovarian atrophy [90]. Benefits of VCD treatment include a more physiological, gradual decrease in gonadal steroid levels and the continued presence of the ovarian tissue [90]. Limitations of VCD are the absence of a comparable model in males (thus prohibiting studies on sex differences) and potential toxic effects on brain function that may be independent of gonadal steroid depletion.

Given current limitations of genetic and toxin models of reproductive aging in studying sex differences in mouse models of AD, we advocate gonadectomy as a relevant and reliable manipulation.

### Gonadal hormone depletion: dissecting a sex difference

If the comparison between gonadectomized males and females reveals a sex difference, further mechanistic dissection can be achieved by determining whether sex chromosomes or organizational effects of hormones (long-lasting effects of hormones that persist in their absence) explain the difference (Figure 3). One strategy to delineate these possibilities is through a genetic approach using the “four core genotypes” (FCG) model. FCG mice produce XX mice with ovaries, XX mice with testes, XY mice with ovaries, and XY mice with testes (reviewed in [91,92]). These mice can be crossed with transgenic mouse models of disease, including AD models. Gonadectomy of all male and female offspring in adulthood enables a complex but precise comparison of whether the observed sex difference is explained by sex chromosomes or organizational effects of gonadal hormones (as reviewed in [91]).

## Mouse models of AD

### Introduction. Using animal models

The necessity of dissecting molecular mechanisms and identifying potential therapies to improve the human condition requires using animal models of disease. A model is only useful, however, if the discoveries generated truly inform us about human disease and potential treatments. Thus, attention to aspects of the AD mouse model that recapitulate clinical manifestations of human AD, such as cognition and its underlying substrates, is essential.

### AD mouse models

While AD is a strictly human disease [101], the generation of genetically modified mice expressing mutations in genes that cause AD has enabled progress in understanding its pathogenesis. Genetic AD results from mutations in genes that regulate the production of Aβ: APP, presenilin 1 (PS1), and presenilin 2 (PS2) (reviewed in [5,101-103]). Most AD mouse models overexpress a mutated form of the human APP gene, a combination of mutated APP and PS1, or a combination of APP, PS1 and P301L (a tau mutation causing frontotemporal dementia) (reviewed in [101,102]). Precise contributors to neural dysfunction, however, are not always clear in the models. For example, in transgenic hAPP mouse models, relative pathogenic contributions of different hAPP processing products are unknown. In addition, proteins such as endogenous APP have normal functions in synaptic physiology and neuronal migration (reviewed in [104,105]) that may be disrupted, further contributing to neural dysfunction.

The development of new mouse models of AD offers the opportunity to optimize paradigms and tackle unanswered questions. For example, models that express hAPP via targeted insertion and from the gene’s own promoter might produce more physiological results. Yet, such “knock-in” models of AD have shown very mild, if any, tractable AD-like disease progression or dysfunction [106-108] to date. Other approaches in current development include modeling sporadic AD, incorporating multifactorial etiologies of AD into models, and integrating other diseases of aging into AD models.

Despite the fact that current mouse models of AD are imperfect representations of the human condition, key features of AD are indeed preserved [101,102] and thus enable meaningful studies using these models. Most notably, transgenic mice show synaptic dysfunction/loss and network disruption, which correlate more closely with cognitive decline, the primary clinical manifestation of AD, compared to other disease measures [6,9].

### Relevant substrates of cognitive decline

The efficacies of therapies, and their relevance to patients and families, depend on whether they can prevent or reverse cognitive decline, the primary clinical manifestation of AD. Thus, its human relevance, combined with the incredible homology between rodent and human memory systems [109], make cognition and its substrates high-yield outcome measures in mouse models of AD. Since the main drivers for cognitive decline are synaptic loss/dysfunction and network disruptions, as discussed above (reviewed in [6,10,40]), these measures, along with their causative agents, are the most directly human-relevant assessments [5,6].

## Review of studies on sex differences in mouse models of AD

### Overview

Sex-based differences in AD exist and comparisons between males and females that model AD are invaluable. Understanding sexual dimorphisms can inform us about what protects one sex or makes the other more vulnerable. Yet, studies that have compared AD-related measures in male versus female mice have reported quite varying results (cognitive differences reviewed in Table 1) making overall conclusions challenging at this time. We believe that the variation in data is probably not random and, in large part, results from the inherently complex comparison of gonadally intact males with gonadally intact, cycling females – a comparison involving multiple layers of biologic effects. Consider the following:

1. If a sex difference exists (or not), it may result from differential activational effects of endogenous gonadal steroids in the male versus the female brain. That is, actions of androgen in the male brain may differ from actions of estrogens or progesterone in the female brain. It is also possible that the sex difference may exist even in the absence of gonadal hormones.

2. Androgen levels remain constant in males while estrogen and progesterone levels fluctuate in females across the estrous cycle. Stages of the estrous cycle influence brain functions of females in diametrically opposing ways [110]. Since female mice cycle synchronously, AD-related outcome measures in females may be biased toward either an estrogen-dominant (proestrous or estrous) [111-113] or a progesterone-dominant (metestrous or diestrous) [111-113] state. Conversely, important hormone-mediated effects in females could be missed if multiple estrous stages are inevitably combined in the experimental group.

3. The ability to detect, repeat, or precisely interpret a sex difference in mouse models of AD can be obscured by points one and two.

In light of these complexities, we review the current literature on sex differences in mouse models of AD – and stress that while comparing gonadally intact males and females is a valuable first step, studies that incorporate human-relevant manipulations to model reproductive aging via gonadal steroid depletion in both sexes are urgently needed.

**Table 1:** Sex Differences in cognition and behavior in mouse models of AD

	**Hormone Status, Age**	**Comparison**	**Cognition & Behavioral Measure and Ref.**	**AD Tg Model**
**Baseline**	Intact, 8 mos (F), 12 mos (M)	Intact: F vs M	F Tg ↑ activity vs F Ntg; M Tg no deficit (OF) [114]	APPswe-PS1dE9
	Intact, 2–15 mos [115], 6–10 mos [121]	Intact: F vs M	F Tg ↓spatial/fear memory at some ages vs F Ntg & M Tg (MWM, PA) [115]	3xTg-AD [115]
			F Tg↔M Tg impairment in novel object recognition (NOR) [115,121]	TASTPM [121]
	Intact, 2–14 mos	Intact: F vs M	F Tg ↓ SAB at 12-14 mos vs F Ntg and M Tg (Y-maze) [116]	3xTg-AD
	Intact, 2–17 mos	Intact: F vs M	F Tg impaired and no change with age (CTA) [127]	APPswe/ PS1dE9
			M Tg increasingly impaired with age (CTA) [127]	
	Intact, 6 mos	Intact: F vs M	F Tg ↓activity/spatial memory/novel arm preference vs F Ntg; M Tg no deficit (OF, MWM, Y-maze) [117]	3xTg-AD
	Intact, 6 mos	Intact: F vs M	F Tg ↑ activity & ↑entries into light vs F Ntg; M Tg vice versa (OF, DLB) [118]	3xTg-AD
	Intact, 3, 9 mos	Intact: F vs M	M Tg ↑activity & ↓SAB vs M Ntg; F Tg no deficit (OF, Y-maze) [119]	APPsw
			F Tg ↓spatial memory vs F Ntg 3 mos; M Tg no deficit (Circular Platform)^**A**,^[119]	
	Intact,16 mos	Intact: F vs M	F Tg↔M Tg impairment in spatial acquisition memory (Barnes Maze) [122]	APPswe/ PS1dE9
	Intact, 3, 9 mos [119], 6 mos [118]	Intact: F vs M	F↔M, Tg↔Ntg, no spatial learning impairments (MWM) [118,119]	APPsw [119]
				3xTg-AD [118]
	Intact, 6, 15 mos	Intact: F vs M	M Tg ↑center time vs F Tg and M Ntg (OF) [120]	3xTg-AD
**Response to Manipulation**	Intact, 8 mos (F), 12 mos (M)	Intact: F vs M	COX-2 overexp ↓SAB in F Tg; no change or impairment in M Tg (Y-maze) [114]	APPswe/ PS1dE9
			COX-2 overexp ↔ novel arm preference^**B**^ in F or M, Tg or Ntg (Y-maze) [114]	
	Intact, 6 mos	Intact: F vs M	Running ↑open arm entries in F Tg & Ntg and ↓ in M Tg & Ntg (EPM^**C**^) [117]	3xTg-AD
	Intact, 6 mos	Intact: F vs M	Running ↑spatial memory/novel arm preference in F Tg vs non-running F Tg; no effect or impairment in M Tg (MWM, Y-maze) [117]	3xTg-AD

Symbols: ↑=increase, ↓=decrease, ↔=no change or difference.

Abbreviations: *F*=Female, *M*=Male, Ntg=non-transgenic, *Tg*=transgenic, mos=months, d=day, *SAB*=spontaneous alternating behavior (thought to reflect working memory), *MWM*=Morris water maze, *OF*=open field, *PA*=passive avoidance, *CTA*=conditioned taste aversion, *NOR*=novel object recognition, *DLB*=dark-light box, *EPM*=elevated plus maze, *COX-2*=cyclooxygenase 2, *overexp*=overexpression.

**A**, Latency to reach escape hole and number of errors.

**B**, Preference for the novel arm in Y-maze thought to reflect spatial recognition memory [114].

**C**, No 3xTg-AD effect was observed in the EPM [117].

### Cognition and Behavior

For the reasons outlined above, it may not be surprising that reports on sex differences between intact male mice compared to intact female mice in AD-relevant cognitive and behavioral tasks vary considerably, even within the same AD model (Table 1). At baseline, females were more impaired than males [114-119], males were more impaired than females [119,120], and the two sexes did not differ [115,118,119,121,122]. There was similar variation in sex differences resulting from genetic or other manipulations in mice that model AD (Table 1). That is: females worsened [114,117], improved [117], or did not differ [114] compared to males. We emphasize that this variability is probably not random, but results from the complexities of differential hormonal actions in male and female brains.

It is curious, however, that intact females are impaired slightly more often than intact males when considering all studies together. While these studies have concluded that female mice are more vulnerable to AD-related deficits, we offer two additional interpretations: in mouse models of AD, 1) female gonadal hormones are more deleterious in female brains than male hormones in male brains or 2) male gonadal hormones are more beneficial in male brains than female hormones in female brains. It will be necessary to further dissect these complex possibilities, beginning by simply depleting gonadal steroids and then comparing males to females. Gonadal steroid depletion in male and female mice will enable us to determine what sex differences exists in a hormonal milieu that is more comparable to the human condition (Figure 3).

**Table 2:** Female studies of cognition & behavior in mouse models of AD

**Hormone/ Regimen**	**Manipulation**	**Timing, Route, Dose**	**Comparison**^**A**^	**Cognition & Behavioral Measure and Ref.**	**AD Tg Model**
**Hormone Depletion**	Gnx 3 mos	N/A	Intact vs Gnx	Gnx ↓ SAB (Y-maze) [162,163]	3xTg-AD
				Gnx ↓ open arm time (EPM) [164]	
	Gnx 3 mos	N/A	Intact vs Gnx	Gnx ↔ spatial memory (MWM) [159]	APP+PS1
	VCD 2-2.5 mos	N/A	Intact vs VCD	VCD ↔ spatial/working memory (RAWM) [160]	APPswe
**Estrogens**	Gnx 3 mo	Immediate s.c. E_2_ (0.025 mg) 90 d	Gnx: Veh vs E_2_	E_2_ ↑ SAB (Y-maze) [162,164]	3xTg-AD
				E_2_ ↓ freezing (FST) [164]	
	Gnx 3 mos	Immediate s.c. PPT (0.25 mg) 90 d	Gnx: Veh vs PPT	PPT ↑ SAB (Y-maze) [162]	3xTg-AD
	Gnx 3 mos	Immediate s.c. DPN (0.25 mg) 90 d	Gnx: Veh vs DPN	DPN ↔ SAB (Y-maze) [162]	3xTg-AD
	Gnx 3 mos	Immediate, 3, & 6 mo later, s.c. E_2_ (0.18 mg)	Gnx vs Gnx + E_2_	E_2_ ↔ spatial memory (MWM) [159]	APP+PS1
	VCD 2-2.5 mos	Immediate s.c. E_2_ (0.36 mg) 90 d	VCD vs VCD+E_2_	E_2_ ↔ spatial/working memory (RAWM) [160]	APPswe
**P**_**4**_**Continuous**	Gnx 3 mos	Immediate s.c. P_4_ (25 mg) 90 d	Gnx: Veh vs P_4_	P_4_ ↔ SAB (Y-maze) [163,164]	3xTg-AD
				P_4_ ↑ open arm time, ↓ freezing (EPM, FST) [164]	
	Gnx 6 mos	Immediate s.c. P_4_ (25 mg) 90+d	Gnx vs Gnx + P_4_	P_4_↔ spatial memory (NPR, MWM) [169]	APPswe+ PSEN1ΔE9
**P**_**4**_**Cyclical**	Gnx 3 mos	Delayed s.c. P_4_ (2.8 mg) 10 d off/on	Gnx: Veh vs P_4_	P_4_ ↔ SAB (Y-maze) [164]	3xTg-AD
				P_4_↑ open arm time, ↓ freezing (EPM, FST) [164]	
**E**_**2**_**+ P**_**4**_**Continuous**	Gnx 3 mos	Immediate s.c. E_2_ (0.025 mg) + P_4_ (25 mg) 90 d	Gnx: Veh vs E_2_+ P_4_	E_2_+ P_4_ ↑ SAB (Y-maze) [163]	3xTg-AD
	Gnx 3 mos	Immediate s.c. E_2_ (0.025 mg) + P_4_(25 mg) 90 d	Gnx: Veh vs E_2_+ P_4_	E_2_+ P_4_ ↔SAB (Y-maze) [164]	3xTg-AD
				E_2_+P_4_ ↑ open arm time, ↓ freezing (FST, EPM) [164]	
**E**_**2**_**+ P**_**4**_**Cyclical**	Gnx 3 mos	Immediate s.c. E_2_ (0.025 mg) 90 d + P_4_ (2.8 mg) 30 d cycles	Gnx: Veh vs E_2_+ P_4_	E_2_+ P_4_ ↑ SAB (Y-maze) [164]	3xTg-AD
				E_2_+P_4_ ↑ open arm time, ↓ freezing (FST, EPM) [164]	
**LH Depletion**	21 mos	I.M. injection of leuprolide followed by depot (7.5 mg/kg)	Intact: Veh vs leuprolide	leuprolide ↑ SAB in aging (Y-maze) [180]	Tg2576
**Testosterone**	Postnatal d1–7	IP injection of T (100 μg/d)	Intact: Veh vs T	T↔SAB (Y-maze) [116]	3xTg-AD

Symbols: ↑=increase, ↓=decrease, ↔=no change.

Abbreviations: *Tg*=transgenic, *mos*=months, *d*=day, *E*_*2*_=estradiol (a type of estrogen), *P*_*4*_=progesterone, *LH*=Leutinizing hormone, *VCD*=4-vinylcyclohexene diepoxide (causes chemically-induced ovarian atrophy), *s.c.*=subcutaneous, *I.M.*=intramuscular, *IP*=intraperitoneal, *SAB*=spontaneous alternating behavior (thought to reflect working memory), *FST*=forced swim test, *RAWM*=radial arm water maze, *NPR*=novel place recognition, *EPM*=elevated plus maze, *PPT*= Propylpyrazole triol (ERα agonist), *DPN*= Diarylpropionitrile (ERβ agonist), *leuprolide*=leuprolide acetate (gonadotropin-releasing hormone agonist).

**A,** All comparisons are between groups of AD Tg mice.

### Aβ, tau, and histopathology

Like cognitive and behavioral data on intact males and females that model AD, there is considerable variation in reports of sex differences in baseline Aβ levels and amyloid plaque deposition: females had higher levels than males [116-118,121,123-132], males had higher levels than females [133,134], and the two sexes did not differ [115,135,136]. In addition, tau levels did not differ in intact males and females [115,123]. Similar variation in sex differences of Aβ levels, amyloid plaque deposition, and tau is observed in response to stress, pharmacologic, and genetic manipulations of mouse models of AD [114,128,131-134,136-139].

As we learn more about the pathophysiology underlying AD, we are coming to appreciate that moderate differences between plaque load or neurofibrillary tangle abundance bear less biologic relevance to cognition than once thought. Nonetheless, levels of plaques and tangles, combined with more toxic assemblies of Aβ and tau should be further assessed in gonadal steroid depleted mice.

### Molecular and Biochemistry

Many studies have examined molecular and biochemical differences between gonadally intact male and female mice that model AD. Among reports are measures of: hAPP processing enzymes and products [123,125,130,133,134,137], corticosteroids [115,120,135], metals [125,130,140,141], immune modulators [131], lipids and their peroxidation products [142], and other factors [128,129,132,143]. All of these measures undoubtedly bear relevance to AD and its pathophysiology. But the significance of the molecular and biochemical sex differences will become clearer with further mechanistic dissection. Do the sex differences persist following depletion of gonadal hormones? If so, how do the molecular and biochemical measures relate to cognitive and behavioral performance in AD models? Factors that correlate well with either protective or detrimental cognitive measures in the absence of gonadal steroids, might lead to important sex-based therapeutic targets in the treatment of AD.

### Survival

Mice that model AD suffer premature mortality [119,120,144-150] in parallel with the human condition. However, whether the striking sexual dimorphism in survival – men with AD progress faster to death than women [41-45] – is recapitulated in mouse models, remains to be determined. Premature mortality has been reported in both sexes [119] but further investigation into this AD-relevant measure is currently lacking. Sex differences in survival bear relevance to the human condition, and may hold promise for revealing novel targets for the development of therapies, if mouse models parallel human epidemiologic findings.

### Conclusions and future directions

While comparing intact male and female mice that model AD is an initial step, we propose that the next step requires modeling human reproductive aging (depleting gonadal steroids via gonadectomy) in mice. We strongly advocate for this research strategy with the following scientific rationales:

1. AD is a disease of aging that, in humans, develops in a gonadal steroid-depleted state; thus, depleting gonadal steroids in mice is a human-relevant manipulation.

2. Endogenous gonadal steroids profoundly impact cognition and brain function (reviewed in [151-156]); thus, removing gonadal steroids in males and females enables a more direct comparison between sexes that is less confounded by “activational” effects of endogenous gonadal steroids.

In the presence of endogenous gonadal steroids, differences between males and females may result from differential actions of androgens in males, or of estrogen/progesterone in females. Further, hormonal effects in females will vary according to the stage in estrous cycle [111-113]. Thus, removing gonadal steroids enables a more precise comparison between the sexes.

## Review of studies in mouse models of AD: females

### Overview

Whether and how reproductive aging or hormone replacement alters vulnerability to AD in females are outstanding questions. Some answers are taking form, but the overall picture remains unclear. Since the WHI and other clinical studies reported negative impacts of HRT on cognition and AD risk [65-67], some mouse studies have begun to determine whether timing, dose, regimen, or route of administration of hormone therapy alters its effects. Key issues in interpreting the results of the mouse studies, which are more fully described in Table 2, are reviewed. Of note, our review is focused on estradiol^c^ (the most biologically active estrogen) and progesterone effects in female mice since these are the principle, systemic gonadal hormones decreased following menopause; but other steroids, including neurosteroids ([180] and reviewed in [157,158]), may also play a role in AD.

### Cognition and behavior

While estradiol facilitates synaptic plasticity and several forms of hippocampal-dependent learning and memory in the adult and aging rodent brain, (reviewed in [151-153]), its role in memory of the diseased brain remains less clear. To date, studies examining hippocampal-dependent spatial learning/memory in AD mouse models find no effects of gonadal hormone depletion [159,160], or estradiol replacement [159,160] in water maze tasks, despite estradiol-mediated decreases in Aβ levels or deposition [161-168]. In parallel, progesterone replacement also failed to alter AD-related impairments in the watermaze [169].

In contrast to the watermaze studies, studies in the 3xTg mouse model show impaired working memory in the Y-maze following gonadectomy [162,163] – an effect that is reversed by estradiol [162-164] but not progesterone replacement [163,164]. The effect of estradiol is mimicked by an ERα but not ERβ agonist [162] and persisted with a combined estradiol and cyclical progesterone regimen [164]. More studies of females are needed in AD mouse models to draw conclusions and determine: whether certain AD models are more sensitive to hormonal effects compared to others (and why), if hormone replacement differentially affects certain cognitive domains and not others in the diseased brain, and ultimately whether hormonal effects on cognition and behavior in female AD model-mice reliably recapitulate human findings.

### Aβ, tau, and histopathology

A large body of evidence, with notable exceptions [136,159,160,170], supports a role for estradiol [161-168,171] and other hormone replacement regimens [164] in decreasing Aβ levels and plaques. However, estrogen-mediated decreases of Aβ, even in its more toxic forms, do not correlate consistently with improved cognition. This striking disconnect, combined with adverse cognitive effects of estrogen replacement in human AD trials [65,66] lead us to speculate that estrogens may be detrimental to substrates of learning and memory in the diseased brain – and that this harmful action negates their beneficial effects on lowering Aβ. Studies are needed to explore this untested hypothesis.

### Molecular and Biochemistry

Consistent with estradiol-mediated decreases in Aβ levels, estradiol treatment suppresses the Aβ producing β-secretase 1 (BACE) [168], and increases the Aβ catabolizing Insulin-degrading enzyme (IDE) [165]. Although this may explain decreased Aβ levels, the conundrum of the hormone’s effect on cognition in the diseased brain remains.

### Survival

Studies have reported survival data in female AD-model mice following gonadal hormone depletion or hormone replacement – and consistently show that estrogens are toxic. Estradiol treatment increased mortality in both intact and hormone-depleted adult female APPswe mice [160]. Interestingly, removal of gonadal steroids before sexual maturity, via gonadectomy, increased mortality in this model [172]; however, when depletion of gonadal steroids was delayed until after sexual maturity, premature mortality did not occur [160]. Since premature mortality in mouse models of AD is closely associated with sudden death from seizures [144-150], the studies suggest that estrogens may increase network excitability leading to seizure-related death in adult, female AD-model mice. In support of this, decreasing brain estrogens through anastrozole in the adult female brain increased survival in 3xTg-AD mice [136].

### Conclusions and future directions

**Figure 6:**
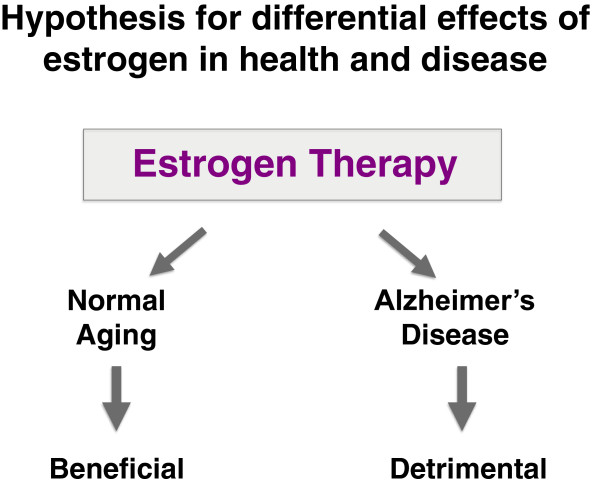
Hypothesis: Estrogen replacement therapy is beneficial in normal aging, but detrimental in AD or to those at risk of developing AD.

The known enhancing effects of estradiol on synaptic plasticity and memory in the normal brain (reviewed in [151-153]), juxtaposed to its conflicting effects on cognition in the diseased brain, lead us to an intriguing hypothesis we wish to put forth: **Estrogens benefit cognition in the normal, aging brain but not in the diseased, AD brain (or in the brain at risk for AD)** (Figure 6). In support of this hypothesis, is data from human clinical trials [65,66] and the following untested rationale. Estrogens increase excitability in the normal brain (reviewed in [151,152]), a process that facilitates normal learning and memory. However, this same action in the hyperexcitable AD-brain could lead to excitotoxicity and memory impairment. Answers to this untested hypothesis could dramatically impact the future of personalized HRT.

## Review of studies in mouse models of AD: males

### Overview

Whether androgens, or other forms of hormone replacement in men with AD are beneficial has yet to be determined in rigorous, double-blinded, prospective, and placebo-controlled clinical trials – but clinical observations [85,173-176] and data from animal studies suggest they may be. Studies of hormone depletion and treatment in male mice that model AD collectively show a beneficial action of androgens on cognition, pathology, and biochemical measures.

### Cognition and behavior

Gonadal steroid depletion, via gonadectomy of male mice that model AD, worsened cognitive impairment [177]. The impairment was reversed by subcutaneous treatment with dihyrdotestosterone (DHT) [177], a metabolite of testosterone. Increasing serum and brain levels of testosterone via genetic knockdown of aromatase [178] also improves cognition in male AD mice. The possibility that androgens may exert long lasting “organizational” effects is supported by a finding that blocking androgen receptors during a critical period of brain differentiation in male 3xTg mice worsens cognition in adulthood [116]. Collectively, these studies (Table 3) show a reproducible, beneficial effect of androgen-related function on cognitive impairments in male mice that model AD.

**Table 3:** Male studies of cognition & behavior in mouse models of AD

**Hormone/ Regimen**	**Manipulation**	**Timing, Route, Dose**	**Comparison**^**A**^	**Cognition & Behavioral Measure and Ref.**	**AD Tg Model**
**Hormone Depletion**	Gnx 3 mos	N/A	Intact vs Gnx	Gnx ↓ SAB (Y-maze) [177]	3xTg-AD
**Flutamide**^**B**^	Postnatal d1-20	IP injection of flutamide (50 mg/kg/d)	Intact: Veh vs Flutamide	Flutamide ↓ SAB (Y-maze) [116]	3xTg-AD
**Genetic Knockdown of Aromatase**^**C**^	None	N/A	Intact: Aromatase^+/−^ vs Aromatase^+/+^	Aromatase^+/−^ ↑ spatial memory (Hole-board) [178]	APP23
**DHT**	Gnx 3 mos	Immediate s.c. DHT (10 mg) 90+d	Gnx: Veh vs DHT	DHT ↑ SAB (Y-maze) [177]	3xTg-AD

Symbols: ↑=increase, ↓=decrease, ↔=no change.

Abbreviations: *Tg*=transgenic, *mos*=months, *d*=day, *Gnx*=gonadectomy, *SAB*=spontaneous alternating behavior (thought to reflect working memory), *IP*=intraperitoneal, *Veh*=Vehicle, *DHT*=dihydrotestosterone.

**A,** All comparisons are between groups of AD Tg mice.

**B**, Flutamide is an androgen receptor antagonist.

**C**, Males with aromatase knockdown have increased endogenous testosterone levels, and decreased brain and sera estradiol levels.

### Aβ, tau, and histopathology

In parallel with protecting against cognitive deficits, androgens also decreased levels of pathogenic proteins and pathology related to AD. While gonadectomy increased Aβ levels and plaque deposition in male AD-model mice [177,179], elevating androgens through hormone replacement [177,179] or genetic knockdown of aromatase [178] decreased these measures. In addition, transient androgen receptor blockade in the neonate [116] curiously increased Aβ accumulation in adulthood, suggesting that androgens can exert organizational effects on propensity toward Aβ pathology. Gonadectomy did not change levels of tau in male 3xTg mice [179], but testosterone decreased tau levels compared to both gonadectomized and intact mice [179].

### Molecular and biochemistry

Consistent with androgen-mediated decreases in Aβ levels, elevating brain and serum testosterone via aromatase knockdown, modified important enzymes (BACE, neprilysin (NEP), and IDE) leading to decreased Aβ production [178]. These data offer an explanation for androgen’s protective actions in lowering levels of Aβ and, ultimately, improving cognition. Although more studies have addressed molecular and biochemical measures in *in vitro*, cell culture systems, they are not reviewed here.

### Survival

No studies to date have examined if androgens, or the lack of, alter survival in male mice that model AD.

### Conclusions and future directions

Though the number of studies is limited, they collectively show a deleterious effect of gonadectomy and a protective action of androgens in male mice that model AD. The protective effect existed in more than one AD model, was achieved using genetic or pharmacologic strategies, and modified a directly relevant measure to clinical AD – cognition. Several important questions arise from these studies. How do androgens modulate Aβ-targeting enzymes and can they protect against cognitive deficits in an Aβ-independent manner? Do androgens signal through receptor-dependent or alternate pathways to achieve protection? Can androgen signaling improve AD-related cognitive deficits in both males and females? If so, can we modify androgen signaling to improve cognition without eliciting masculinizing effects? These and other lines of androgen-focused investigation may hold promise for treating the human condition.

## Where do we go from here?

### Research strategies

Given our evolving tools and knowledge base, we put forth the following suggestions for future research in sex- and hormone-based studies in AD mouse models. First, since gonadal steroid depletion is an inextricable aspect of human, but not mouse, aging, it should be vigorously incorporated into our study of AD and other disease models of aging. Gonadal steroid depletion through gonadectomy is a human-relevant manipulation that models the hormone environment in which AD develops and enables clearer approaches to comparing males and females, understanding reproductive aging, and testing effects of hormone replacement. Second, when using mouse models of AD, measures that relate closely to clinical AD, and thus directly reflect human-relevant outcomes, should be included in studies. This means expanding the focus of mouse model research to include cognition, behavior, and measures closely correlated with these, such as synaptic and network function. Third, because clear sex differences exist in human AD epidemiology, specifically in progression to death, it is important to look at survival and related measures in our mouse models. Finally, in light of past, present, and future clinical trials, we should continue lines of research studying how the loss and replacement of hormones affect each sex in models of AD.

### Outlook

As we learn more about AD itself, how it manifests differently in men compared to women, and how hormones modify its risk, we must simultaneously recast our questions to reflect high-yield and human-relevant research strategies – with the goal of achieving biomedical discoveries that improve the human condition. This is not an easy task, as it requires merging the complex, emerging field of sex and hormone biology with the study of a complex, devastating disease of aging, AD. We believe this task is not insurmountable, and if taken on mindfully, may reveal novel targets for defeating the disease.

## Endnotes

^**a**^Sex is biological classification of living beings as male or female and is used in this review to describe both humans and mice. Gender, a term that is only appropriate when applied to humans, is a cultural expression of sex, shaped by environment and experience.

^**b**^This review of sex differences in the epidemiology of AD includes large populations in which the effect of ApoE4, a genetic risk factor that increases AD risk in women, is not specifically examined.

^**c**^Estradiol is the most biologically active form of estrogen that circulates in high levels in the body prior to the menopause in women. Most studies in animal models use estradiol for hormone treatment. In contrast, clinical studies in humans have used hormone replacement paradigms that include other estrogenic steroids.

^**d**^During the publication process of our review, an observational study showing an association between hormone therapy use started within 5 years of menopause and decreased AD risk was released (H. Shao et al. Hormone therapy and Alzheimer disease dementia: New findings from the Cache County Study. Neurology 79, 1846). Though the findings are highly intriguing in light of the critical window hypothesis, caution must be exercised when interpreting observational studies due to their inherent limitations. Ultimately this study approach can only show associations and not causal links. Whether hormone therapy reduces the risk of AD when given during a critical window will need to be determined through the gold-standard of clinical research – randomized, double-blind, placebo-controlled trials.

## Competing interest

The authors declares that they have no competing interest.

## Authors’ contributions

DD, LB and KW reviewed the literature. DD and LB wrote the review. All authors read and approved the final manuscript.

## Authors’ information

Dena B Dubal, MD, PhD is an Assistant Professor of Neurology and Director of the Laboratory for Neuroscience and Aging Research. She holds the David Coulter Endowed Chair in Aging and Neurodegeneration at UCSF. She is a physician-scientist with a clinical and research focus on intersections between aging and neurodegeneration.

Lauren Broestl is a Research Associate in the department of Neurology at UCSF.

Kurtresha Worden is a Research Associate in the department of Neurology at UCSF.
